# The incidence of distal radius fractures in a Swedish pediatric population - an observational cohort study of 90 970 individual fractures

**DOI:** 10.1186/s12891-021-04410-6

**Published:** 2021-06-19

**Authors:** Hanna Südow, Cecilia Mellstrand Navarro

**Affiliations:** 1 Department of Clinical Science and Education, Södersjukhuset Karolinska Institutet, SE-118 83 Stockholm, Sweden; 2 Department of Orthopeadics, Södersjukhuset Hospital, Sjukhusbacken 10, SE-118 46 Stockholm, Sweden; 3 Department of Hand Surgery, Södersjukhuset Hospital, Stockholm, Sweden

**Keywords:** Pediatric fracture, distal radius fracture, epidemiology, seasonal variations, trends

## Abstract

**Background:**

40–50 % of all boys and 30–40 % of girls suffer from at least one fracture during childhood. A quarter of these fractures affects the wrist, making it the worst affected part of the body. Children often sustain the injury during play or sport activities. There has been a lifestyle change among European children during the last decades, and there is reason to believe that fracture incidence is changing.

**Methods:**

For the purpose of this observational cohort study registry data was retrieved from the Swedish National Patient Register for all pediatric patients registered with a distal radius fracture during the period 2005–2013. Incidence rates were calculated for each year using data from Statistic Sweden on population size by age and gender.

**Results:**

90 970 distal radius fractures were identified. The mean age at the time of fracture was 10 years. In ages 10–17 the proportion of male patients was significantly larger. Seasonal variations were detected with peak incidences in May and September. A decreasing total fracture incidence was observed during the study period.

**Conclusions:**

The incidence of distal radius fractures in a population 0–17 years in Sweden was higher among male than in female patients. The incidence was lower in 2008–2013 as compared to 2005. Further studies are necessary to reveal if the incidence will continue to decrease.

## Background

Fractures are common injuries among children. Nearly 40–50 % of all boys and 30–40 % of girls suffer from at least one fracture during childhood [1, 2]. Wrist fractures represent 25 % of all fractures in children [2, 3]. The fracture most commonly occurs when the child falls on an extended arm [2, 4, 5]. Falling while playing in a monkey bar or a trampoline are two frequently reported trauma mechanisms for younger children while the teenagers tend to sustain fractures during sports [6]. The incidence has been reported to peak during early stages of puberty [7–9] when the volumetric bone mineral density is relatively low [7].

The injury is often benign and most children will recover without any major sequelae [10]. A majority of cases are treated non-surgically with a bandage or a forearm cast and sometimes with a cast that immobilizes the elbow, depending on patient age and the character of the fracture [10]. In case of fracture patterns too displaced to be tolerated, closed reduction with or without surgical treatment with percutaneous pinning is recommended [11]. Treatment with open reduction and internal fixation is uncommon in the pediatric population [10].

There is reason to believe that there has been a lifestyle change among European children during the last decades, due to a continuous introduction of new technical devices to a broad population. There are reports describing a decrease in time that children and teenagers spend on physical activities [12]. How these lifestyle changes affect children’s health in general, and more specifically distal radius fracture incidence is largely unknown. Publications presenting fracture incidence in pediatric populations have shown an increasing trend for many decades [3, 13, 14]. A study in a British setting reports data from 2007 to 2014 and showed no change in incidence of distal radius fractures during that period [15].

Most of the available data regarding radius fracture incidence in children describes what has happened over decades and are limited to small populations or reports from regional findings. Knowledge is sparse regarding the fracture incidence development in the new and rapidly developing millennium.

The aim of this nationwide registry study was to investigate the incidence of distal radius fractures in the pediatric population in Sweden during 2005–2013.

## Methods

This is a descriptive observational cohort study analyzing Swedish nationwide registry data from 2005 to 2013. The Swedish National Patient Register (NPR) contains data from mandatory registrations of all inpatient care since 1987, and outpatient care since 2001, and is kept by the Swedish National Board of Health and Welfare under the Ministry of Health and Social Affairs [16]. Diagnoses are registered through the Swedish version of the International statistical classification of diseases and related health problems 10th revision (ICD-10-SE) code system. The coverage of the Swedish NPR is high and data has been reported to be valid since 2005 [17].

Data were retrieved for all registrations of distal radius fractures in pediatric patients during the period 2005 through 2013. We chose 2005 as the study start to avoid possible low coverage in the registry during its first years of activity. Data was ordered from the NPR in 2014 thus limiting the study period to the end of 2013. Patients were identified by the occurrence of the ICD-10-SE codes S52.5 fracture to the distal radius or S52.6 fracture to the distal part of radius and ulna. The study population were all individuals 0–17 years at the day of diagnosis.

A fracture event was defined as the first time the diagnosis S52.5 or S52.6 appeared in the register. If the diagnosis code S52.5 or S52.6 reappeared after a period of at least 18 months without any registrations of a distal radius or ulna fracture, it was considered a re-fracture, and counted as a fracture event. Simultaneous bilateral fracture or repetitive fracture within 18 months were counted as only one fracture. All individuals were sorted into four age groups: 0–6 years, 7–10 years, 11–14 years, 15–17 years.

Data on population size was collected from Statistics Sweden [18] including numbers of inhabitants in one-year age groups broken down by sex as for November 1st every year 2005 through 2013.

### Ethics

Ethical permission was retrieved from the Swedish Ethical Review Authority, reference number 2013/105 − 31/2, 2014/1041-32, 2017/611 − 32. No patients were asked for permission to participate and no personal identifications were retrieved. All data is used on population basis to minimize the intrusion of integrity.

### Statistics

The statistical software used was IBM SPSS Statistics, version 23 and 25 for Windows. Continuous variables were presented as means. Proportions were presented as percentages and were compared with Chi^2−^test. Annual incidence was calculated as the number of fracture events divided by the population at risk and presented as incidence per 10 000 person years. Incidences were calculated for the entire study population and separately for each age category. Fishers test were used to calculate confidence intervals (C.I) for incidence rates. A Poisson regression model adjusting for age group, gender and population at risk was performed, and results are presented as crude and adjusted measures, with a 95 % C.I and according p-value.

## Results

A total number of 90,970 distal radius fractures in patients aged 0 to < 18 years in Sweden were registered during the study period. The mean age at the time for fracture was 10 years (males: 10.73, females 9.33) years and the median 11 (males: 11, females:10) years.

The proportion of male patients in the total population was 60.3 %. The distribution between genders was equal in age groups 0–10 years, but the proportion of male patients was higher in patients 11–17 years (p < 0.001).

**Fig. 1:**
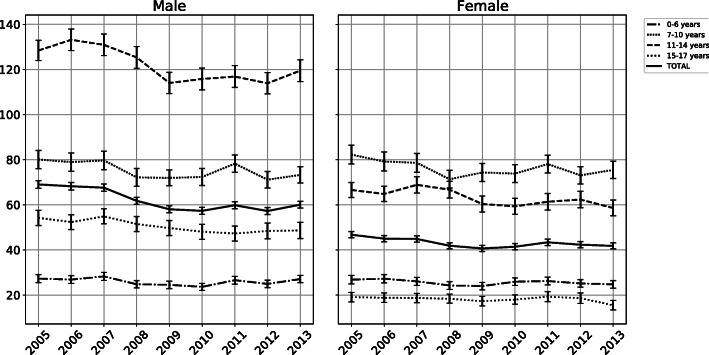
The incidence rates of a distal radius fracture per 10 000 person years in Sweden during 2005 - 2013 according to registrations in the Swedish Patient Registry, presented per age group and gender. Error bars indicating the 95% Confidence Interval

The incidence rate over the whole time period was 52.9/10 000 person years. Male patients aged 11–14 had the highest incidence rate (113–133/10 000 person years). Females fifteen years or older had the lowest incidence rate followed by children 0–6 years (Table 1). The incidence rate differed between age groups and gender during all the studied years (Fig. 1).

**Table 1:** The incidence rates of a distal radius fracture in Sweden during 2005 - 2013 according to registrations in the Swedish Patient Registry presented as incidence rate per 10 000 person years per year and age group. 95% Confidence intervals from Fisher’s exact test

Year	Type	0-6 years			7-10 years			11-14 years			15-17 years			TOTAL		
		Male	Female	Subtotal	Male	Female	Subtotal	Male	Female	Subtotal	Male	Female	Subtotal	Male	Female	Total
2005	n	933	870	1803	1601	1560	3161	3221	1588	4809	1040	346	1386	6795	4364	11159
	Population at risk	341619	324214	665833	199960	189678	389638	250826	238515	489341	191832	181339	373171	984237	933746	1917983
	Incidence Rate	27.31	26.83	27.08	80.07	82.24	81.13	128.42	66.58	98.28	54.21	19.08	37.14	69.04	46.74	58.18
	95% CI	25.6-29.1	25.1-28.7	25.8-28.4	76.2-84.1	78.2-86.4	78.3-84.0	124.0-132.9	63.3-69.9	95.5-101.1	51.0-57.6	17.1-21.2	35.2-39.1	67.4-70.7	45.4-48.1	57.1-59.3
2006	n	945	909	1854	1529	1458	2987	3204	1485	4689	1033	351	1384	6711	4203	10914
	Population at risk	351806	333806	685612	193689	184080	377769	240616	229092	469708	197413	186641	384054	983524	933619	1917143
	Incidence Rate	26.86	27.23	27.04	78.94	79.20	79.07	133.16	64.82	99.83	52.33	18.81	36.04	68.23	45.02	56.93
	95% CI	25.2-28.6	25.5-29.1	25.8-28.3	75.0-83.0	75.2-83.4	76.3-82.0	128.6-137.9	61.6-68.2	97.0-102.7	49.2-55.6	16.9-20.9	34.2-38.0	66.6-69.9	43.7-46.4	55.9-58.0
2007	n	1020	893	1913	1540	1437	2977	2977	1496	4473	1100	353	1453	6637	4179	10816
	Population at risk	360743	342599	703342	193259	182764	376023	227397	217229	444626	200424	189024	389448	981823	931616	1913439
	Incidence Rate	28.27	26.07	27.20	79.69	78.63	79.17	130.92	68.87	100.60	54.88	18.67	37.31	67.60	44.86	56.53
	95% CI	26.6-30.1	24.4-27.8	26.0-28.5	75.8-83.8	74.6-82.8	76.4-82.1	126.3-135.7	65.4-72.5	97.7-103.6	51.7-58.2	16.8-20.7	35.4-39.3	65.6-69.2	43.5-46.2	55.5-57.6
2008	n	921	855	1776	1409	1316	2725	2707	1372	4079	1011	341	1352	6048	3884	9932
	Population at risk	371606	352263	723869	195195	184434	379629	215951	205596	421547	196336	185559	381895	979088	927852	1906940
	Incidence Rate	24.78	24.27	24.53	72.18	71.35	71.78	125.35	66.73	96.76	51.49	18.38	35.40	61.77	41.86	52.08
	95% CI	23.2-26.4	22.7-26.0	23.4-25.7	68.5-76.1	67.6-75.3	69.1-74.5	120.7-130.2	63.3-70.4	93.8-99.8	48.4-54.8	16.5-20.4	33.5-37.3	60.2-63.3	40.6-43.2	51.1-53.1
2009	n	933	863	1796	1433	1406	2839	2356	1182	3538	946	313	1259	5668	3764	9432
	Population at risk	380441	360113	740554	199256	189135	388391	206631	195831	402462	190396	180755	371151	976724	925834	1902558
	Incidence Rate	24.52	23.96	24.25	71.92	74.34	73.10	114.02	60.36	87.91	49.69	17.32	33.92	58.03	40.66	49.58
	95% CI	23.0-26.2	22.4-25.6	23.1-25.4	68.2-75.4	7.05-78.3	70.4-75.8	109.5-118.7	57.0-63.9	85.0-90.9	46.6-53.0	15.5-19.4	32.1-35.8	56.5-59.6	39.4-42.0	48.6-50.6
2010	n	919	954	1873	1486	1439	2925	2318	1127	3445	873	310	1183	5596	3830	9426
	Population at risk	388865	367816	756681	205379	194861	400240	200174	189957	390131	181588	172164	353752	976006	924798	1900804
	Incidence Rate	23.63	25.94	24.75	72.35	73.85	73.08	115.80	59.33	88.30	48.08	18.01	33.44	57.34	41.41	49.59
	95% CI	22.1-25.2	24.3-27.6	23.6-25-9	68.7-76.1	70.1-77.8	70.5-75.8	111.1-120.6	55.9-62.9	85.4-91.3	44.9-51.4	16.1-20.1	31.6-35.4	55.8-58.9	40.1-42.8	48.6-50.6
2011	n	1049	979	2028	1652	1564	3216	2329	1155	3484	813	313	1126	5843	4011	9854
	Population at risk	394431	373474	767905	211114	200362	411476	199301	188247	387548	171952	162410	334362	976798	924493	1901291
	Incidence Rate	26.60	26.21	26.41	78.25	78.06	78.16	116.86	61.36	89.90	47.28	19.27	33.68	59.82	43.39	51.83
	95% CI	25.0-28.3	24.6-27.9	25.3-27.6	74.5-82.1	74.2-82.0	75.5-80.9	112.2-121.7	57.9-65.0	86.9-92.9	44.1-50.6	17.2-21.5	31.7-35.7	58.3-61.4	42.1-44.8	50.8-52.9
2012	n	1000	953	1953	1541	1505	3046	2288	1182	3470	788	284	1072	5617	3924	9541
	Population at risk	400763	378992	779755	216646	205946	422592	200916	189674	390590	162699	152686	315385	981024	927298	1908322
	Incidence Rate	24.95	25.15	25.05	71.13	73.08	72.08	113.88	62.32	88.84	48.43	18.60	33.99	57.26	42.32	50.00
	95% CI	23.4-26.6	23.6-26.8	24.0-26.2	67.6-74.8	69.4-76.9	69.5-74.7	119.3-118.6	58.8-66.0	85.9-91.9	45.1-51.9	16.5-20.9	32.0-36.1	55.8-58.8	41.0-43.7	49.0-51.0
2013	n	1099	949	2048	1641	1604	3245	2462	1144	3606	768	229	997	5970	3926	9896
	Population at risk	405818	383656	789474	223990	212490	436480	206046	195250	401296	157875	147832	305707	993729	939228	1932957
	Incidence Rate	27.08	24.74	25.94	73.26	75.49	74.34	119.49	58.59	89.86	48.65	15.49	32.61	60.08	41.80	51.20
	95% CI	25.5-28.7	23.2-26.4	24.8-27.1	69.8-76.9	71.8-79.3	71.8-77.0	114.8-124.3	55.3-62.1	87.0-92.8	45.3-52.3	13.6-17.6	30.6-34.7	58.6-61.6	40.5-43.1	50.2-52.2

**Fig. 2:**
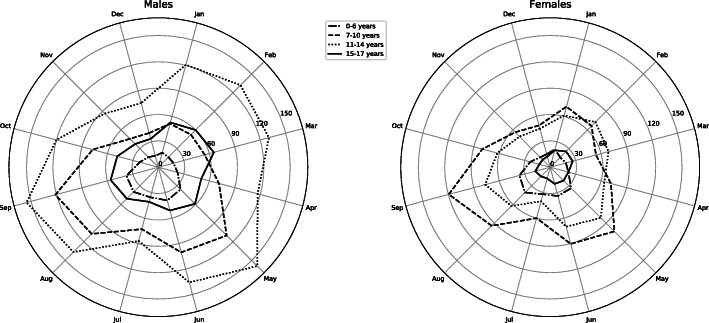
Seasonal variation of incidence rates of a distal radius fracture in different age groups in a polar plot, according to data from the Swedish Patient Registry. Incidence rate is shown per 10000 person years 2005-2013

**Fig. 3:**
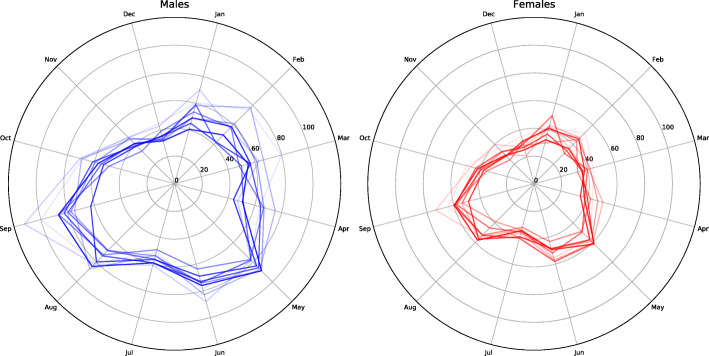
Incidence rates of a distal radius fracture per 10000 person years over the study period according to data from the Swedish Patient Registry. A polar plot is presented for both genders with one line for each year. Opacity in the lines increases from 2005 to 2013

A significant variation over the year was detected (p > 0.001) with peaks in May 68,7/10 000 person years (95 % C.I 69.6–72.4) and September 73,2/10 000 person years (95 % C.I 71.8–74.7) fracture/ 10 000 person years (Figs. 2 and 3). The lowest incidence was found during December at 32,1/10 000 person years (95 % C.I 31.2–33.1).

The incidence as investigated in a Poisson regression model was lower each year 2008–2013 as compared to 2005 (p < 0.001) (Table 2).

**Table 2:** The incidence rate of a distal radius fracture in Sweden during 2005 - 2013 according to registrations in the Swedish Patient Registry. A Poisson regression model illustrates the development of fracture incidence expressed as a relative risk (RR) adjusted for age and gender

		Population at risk	Number of fractures	Incidence rate per 10.000	Univariable		Multivariable Adjusted for all variables*	
					RR	95% CI (***p***-value)	RR	95% CI (***p***-value)
**Year**	**2005**	1917983	11159	58.2	Reference		Reference	
	**2006**	1917143	10914	56.9	0.978	0.953-1.004 (0.099)	0.993	0.967-1.019 (0.592)
	**2007**	1913439	10816	56.5	0.969	0.944-0.995 (0.021)	0.999	0.972-1.026 (0.917)
	**2008**	1906940	9932	52.1	0.890	0.866-0.914 (<0.001)	0.927	0.902-0.953 (<0.001)
	**2009**	1902558	9432	49.6	0.845	0.822-0.869 (<0.001)	0.886	0.862-0.911 (<0.001)
	**2010**	1900804	9426	49.6	0.845	0.822-0.868 (<0.001)	0.886	0.861-0.911 (<0.001)
	**2011**	1901291	9854	51.8	0.883	0.859-0.907 (<0.001)	0.921	0.896-0.947 (<0.001)
	**2012**	1908322	9541	50.0	0.855	0.832-0.879 (<0.001)	0.883	0.859-0.908 (<0.001)
	**2013**	1932957	9896	51.2	0.887	0.863-0.911 (<0.001)	0.899	0.874-0.923 (<0.001)
**Gender**	**Female**	8368484	49107	58.7	Reference		Reference	
	**Male**	8832953	74835	84.7	1.521	1.501-1.541 (<0.001)	1.445	1.425-1.466 (<0.001)
**Age**	**0-6 y**	6592498	22434	34.0	Reference		Reference	
	**7-10 y**	3578632	37429	104.6	1.591	1.561-1.622 (<0.001)	3.193	2.971-3.432 (<0.001)
	**11-14 y**	3820328	49408	129.3	2.088	2.051-2.127 (<0.001)	3.963	3.708-4.235 (<0.001)
	**15-17 y**	3216478	14671	45.6	0.658	0.642-0.674 (<0.001)	1.432	1.320-1.552 (<0.001)

## Discussion

This observational cohort study analyzing Swedish nationwide registry data describes the gender and age distribution as well as incidence rates of distal radius fractures during the period 2005–2013.

Our results confirm the findings of previous authors that distal radius fractures are more common in boys than in girls [15]. However, the incidence rates encountered in our data differed somewhat from that previously presented. In a study from a United Kingdom setting, the incidences were lower than in our study population. Exact comparisons are difficult since we have presented our study populations in different age groups. Differences in fracture incidence between countries may be explained by different cultures, with different playing habits, preventive measures and upbringing conditions [2]. Biological differences may also be present, as is the case in an elderly population where the Scandinavian population is known to be more prone to osteoporotic fractures [19], and hence subject to a high incidence of distal radius fractures. To the best of our knowledge, there is no such differences in predisposing biological factors in the growing skeleton.

In a Dutch study, the incidence rates in the corresponding age groups were approximately similar to our findings [13]. The incidence of distal radius fractures was shown to increase from 1997 to 2007. In contrast, our data suggest a decrease in incidence rates over our study period. We speculate that an extended study period of the Dutch material may reveal a similar slow-down or decrease in fracture incidence that we found. Another speculation may be that an extension of our study period might reveal that our findings of a lowered incidence was a temporary dip in an increasing overall tendency. Future studies are needed to confirm these suggestions.

Jerrhag et al. also present data of an increasing incidence 1999–2010 in a southern Swedish region. They have presented their results as mean annual change, thus not making it possible to discern a possible change in increase at the end of their study period [20].

In nationwide study from South Korea 2005–2009, Park et al. found a higher incidence rate than ours of 80/10 000 person years. In agreement with our study findings, they report a decreasing trend in the late part of the study period [21].

In Germany Körner et al. studied the change in pediatric upper extremity fractures 2002–2017 and reported only a slight change in incidence rate during the study period [22].

There is data that supports that physical and mental wellbeing in children is associated with physical activity [12, 23]. The association between physical activity and occurrence of fractures however is debated: In a study from the United Kingdom, Clark et al. (2008) suggested a positive association between a high level of physical activity and fracture risk in children [24]. In a Swedish population there was no increase in fracture risk in a long-term moderate exercise intervention program among schoolchildren [25]. The influence of physical exercise on fracture risk may act as a protective factor, if one believes that physical activity strengthens the bone structure and improves balance and coordination. It may also counteract childhood obesity and overweight that have been described to be associated with an increased risk of forearm fractures [26, 27]. However, there is reason to believe that exposure to bicycling, tree climbing, monkey bars and trampolines would produce more fractures than computer games or similar digital entertainment. We therefore speculate that the decrease in incidence that we discern in our material may represent a change in injury patterns secondary to life-style changes in children. What long term effect this may have on children’s health and future risk for pathology is yet to be investigated.

Some limitations to this study are admitted. First and foremost, this is a registry study on a population level meaning that details at the individual level are not available. The results rely on the ICD-10-SE code of diagnosis registrations and no details on fracture type, fracture pattern or coinciding observations can be obtained or analyzed. The registry design, on the other hand, offers a nationwide and large study population during a long time providing a great amount of fracture data. The age groups were constructed to separate different populations with different activity patterns. The cut off of different age groups could have been set differently. We believe that all registries may have problems with a low coverage during the first years of registrations which explain our study period started in 2005 even if data was available in the registry from 2001. Another selection of study period could have been more appropriate.

## Conclusions

The incidence of distal radius fractures in the Swedish population 0–17 years of age was lower in 2008–2013 as compared to 2005. Further studies are necessary to reveal if the incidence will continue to decrease. The incidence of distal radius fractures was higher among male than in female patients. There are seasonal variations in distal radius incidences with different patterns in different age groups.

## Data Availability

The material could be retrieved from the corresponding author upon reasonable request.

## Abbreviations

C.I: Confidence intervals
ICD-10-SE: Swedish version of the International statistical classification of diseases and related health problems 10th revision
NPR: The Swedish National Patient Register
